# Thomas on Diseases of Women

**Published:** 1874-11

**Authors:** 


### Bibliographical.

A Practical Treatise on the Diseases of Women. By T. Gaillard Thomas, M. D., Professor of Obstetrics and Diseases of Women and Children, in the College of Physicians and Surgeons, New York, etc., etc. Fourth edition, thoroughly revised, with 191 illustrations on wood. Philadelphia: Henry C. Lea, 1874. Octavo, pp. 801. Cloth, $5; leather, $6. Sold in Atlanta by M. Lynch.

It falls to the lot of few medical authors of the present day to achieve so distinguished success as Dr. Thomas has done in the work before us. The fact that three large editions have been exhausted in the short space of five years, coupled with the further fact that the work has been translated into the German, and is already being rendered into French and Italian, stamps its merit beyond possible question or cavil. To us, of the South, it is a matter of especial pride that we are enabled to claim the distinguished author, not only as an American, but likewise as a native son of our own genial clime.

So familiar is the work of Dr. Thomas to our readers, it would be out of place to attempt now any extended review of it; but so rapidly progressive is the branch of medical science of which it treats, in the present epoch, there will be many who will find it to their advantage to throw aside their old copies and purchase the new. We shall, therefore, endeavor to point out some of the additions and modifications of the new edition, that such may judge of the expediency of the exchange.

Both in his preface and the text of the volume the author insists upon the very great advantage to be gained by employing Sims' duck-bill speculum in the semi-prone position. "He looks upon the introduction of the lateral method of speculum examination as a great advance in gynæcology; he regards it as a method which puts him who practices it upon a decided vantage ground over him who employs the dorsal method; and he confidently looks forward to the day when the great superiority of the levator perinei speculum will cause it to supersede all others."

The semi-prone posture, as it has been called, first suggested, we think, by Sir James Simpson, certainly offers very great advantages over the dorsal decubitus in almost all manipulations about the uterus, either with or without the speculum, whether manual or instrumental. In this posture the pelvic contents tend to gravitate forwards and upwards towards the umbilicus, and the vagina, when opened, becomes inflated with air, giving ample view of its walls as well as the os and cervix; moreover, the uterus being relieved from the pressure of the bladder and superincumbent mass of intestines, becomes very mobile, and can be readily inclined at will, with the use of a tenaculum, sound or forcep. To gain these advantages, however, we have not found it necessary to employ always the Sims' speculum; indeed, we often find a decided inconvenience in its use, because an assistant is required to manage the speculum, while one hand of the operator must be employed in using the depressor. Both of the inconveniences mentioned are readily overcome if we substitute Storer's modification of the Cusco speculum; the skillful use of which enables us to retract the perineum and recto-vaginal septum on the one hand, and press forward the anterior vaginal wall and bladder upon the other hand; and not only so, but a dextrous movement of the blades will enable us to alter the position of the cervix in a very satisfactory way, whilst the speculum is self-retaining, and both hands of the manipulator are free for other uses. If it be desired to fix the os in any desired position, it may be readily done with Nott's little hooked wire secured upon the rim of the speculum, or by the tenaculum forceps in the hand.

The silver, tubular speculum of Meigs, or Ferguson's glass tube, may likewise be employed with decided advantage in the semi-prone posture, and the introduction of almost all forms of pessaries is also facilitated by the position.

We find the following among other additions to the table of contents: Simons' method of rectal exploration; hydrocele of the inguinal canal; hyperæsthesia of the vulva; irritable urethral caruncle; urethral venous angioma; prolapsus urethra; tumors of the vulva; Simon's operation for vesico-vaginal fistula; granular and cystic degenerations of the cervix uteri; perineoraphy; Emmet's operation of elytrorraphy; Thomas' operation of narrowing the vagina; Thomas' operation for inversion of uterus; cysto-fibromata; sarcoma of the uterus; ovarian dysmenorrhœa.

Under the head of vesico-vaginal fistula, the German method of Simon is described at length. It varies in many particulars from the proceeding of our own countryman, Sims, but we are unable to perceive any material advantage which it offers over the latter.

In the treatment of chronic corporeal endometritis the author, by a word, condemns the use made by Atthill, of Dublin, of fuming nitric acid to the endo-metrium—a practice which we have found serviceable.

For the local treatment of areolar hyperplasia, our author recommends the compound tincture of iodine, with a dressing of glycerine, and remarks: "Mild and lacking in vigor as this course may appear, let any one test it side by side with the plan of using the acid nitrate of mercury, potassa fusa, and potassa cum. calce, and the actual cautery; of swabbing out the uterine cavity with chemically pure nitric acid, or of leaving a piece of solid nitrate of silver to melt within it; and, unless his experience greatly differ from mine, he will feel that in the former he has reached a resting place for his faith in the treatment of the most important of all the forms of uterine disease. He will see proof daily spring up before him that his capacity for benefiting his patients has greatly increased, while his liability to injuring them has as markedly diminished."

The chapter upon cancer of the uterus is re-written and greatly improved. Mention is made of Simon's method of partial removal by the scoop, and the subject closed with the following admirable *resume* of the indications for treatment:

1. Secure cleanliness, prevention of fetor, and diminution of hemorrhage and pain by the free use of tepid vaginal injections of antiseptic and astringent character, such as the following:

| | |
|---|---|
| ℞.—Acidi carbolici, | ℥ iiss. |
| Glycerinæ, | Oj. |
| Aluminis sulphatis, | ℥ xiv. |
| Morphiæ sulphatis, | gr. xvi. |

M. Sig.—Add one tablespoonful to two quarts of tepid water, and use as a vaginal injection—morning and evening.

2. Give an abundance of food *which the system can appropriate*, at regular intervals, bearing in mind that nutrition consists in the introduction into the blood, not into the stomach alone, of nutrient materials.

3. Do not indulge in, what appears to be to a certain order of medical mind, the grim pleasure of making a fatal prognosis; as long as possible let the patient enjoy the pleasures of hope. It is not the duty of the physician to hold constantly before her eyes the gloomy picture of a speedy and certain death which he is powerless to avert. No deception should be practiced, and none need be, for these patients always suspect the truth, and do not seek to be informed. Immediate relatives should have the facts plainly stated to them.

4. Quiet pain by the systematic use of opium or one of its alkaloids. The use of the hypodermic syringe at a fixed hour every day is the most certain, and frequently the most agreeable plan.

5. If possible, remove the diseased part by electro-cautery.

6. If complete removal be impossible, and the vagina, bladder, rectum, or pelvic tissues be involved, avoid surgical interference entirely.

7. If the disease be confined to the uterus, and complete removal be impossible, practice partial removal or destruction of the growth by galvano-cautery, the scissors, scoop, or curette, or by actual cautery, fuming nitric acid, the gas jet cautery or potassa cum calce.

Of the treatment of amenorrhœa, dependent upon absence of the uterus, attended as the condition often is, by most distressing and alarming symptoms, we think it ought *not* to be said at the present day: "If the uterus be found to be absent, all that can be done will be to abstract a sufficient amount of blood from the arm by vene-section, if necessary, to relieve the urgent symptoms attending each epoch." The retention of this sentence in the new edition is, we presume, an oversight of the author.

The chapter upon ovariotomy has been thoroughly revised and materially extended. Of normal ovariotomy, our author remarks:

"Like every other bold innovation in medicine, this will have to run the gauntlet of prejudice, and stand the test of experience. It is too young as yet to be decided upon, and is unquestionably a procedure which may be greatly abused. Nevertheless, I freely commit myself to the opinion that it has a future before it which will be rich in good results. Since the publication of Dr. Battey's essay, I have met in the Woman's Hospital with one case which I felt demanded a resort to it, and, with the full indorsement of my colleagues, Sims, Peaslee, Metcalfe and Fordyce Barker, it was safely performed. Three months have since elapsed, a period too short to warrant a report of the case, but I may here say that the patient's condition has been greatly improved."

Vaginal ovariotomy receives due attention, the author's case is fully detailed, and the essential particulars of those operated upon by Gilmore, of Alabama, and Battey, of Georgia. We think it may be confidently asserted that the use of Bozeman's securing apparatus, with the repeated changes of position of the patient, as described by our author, are not only exceedingly inconvenient, but wholly unnecessary. Our own case was operated in the usual semi-prone position of Simpson. When it is desired to counteract the forward and upward gravitation of the pelvic contents, it suffices to compress the abdomen a little by the hands of an assistant, without change of position of the patient. One who is competent for the execution of such an operation should be able to make the incission in the median line notwithstanding the obliquity of the position.

We think that Dr. Thomas does not claim too much for this operation when he says: "I feel sure that this procedure will, when its merits have been fairly tested, occupy an important place in the treatment of ovarian cysts.

A number of new illustrations have been added, and the "getting up" of the volume, like the publishing house from which it emanates, is suggestive of thoroughness and durability.

